# Improved multi-layer wavelet transform and blind source separation based ECG artifacts removal algorithm from the sEMG signal: in the case of upper limbs

**DOI:** 10.3389/fbioe.2024.1367929

**Published:** 2024-05-20

**Authors:** Wei Lu, Dongliang Gong, Xue Xue, Lifu Gao

**Affiliations:** ^1^ School of Management, Fujian University of Technology, Fuzhou, China; ^2^ School of Mechanical and Automotive Engineering, Fujian University of Technology, Fuzhou, China; ^3^ School of Electronic, Electrical Engineering and Physics, Fujian University of Technology, Fuzhou, China; ^4^ Institute of Intelligent Machines, Hefei Institutes of Physical Science, Chinese Academy of Sciences, Hefei, China

**Keywords:** surface electromyography, electromyogram, artifact removal, wavelet transform, blind source separation, fuzzy entropy

## Abstract

**Introduction:** Surface electromyogram (sEMG) signals have been widely used in human upper limb force estimation and motion intention recognition. However, the electrocardiogram(ECG) artifact generated by the beating of the heart is a major factor that reduces the quality of the EMG signal when recording the sEMG signal from the muscle close to the heart. sEMG signals contaminated by ECG artifacts are difficult to be understood correctly. The objective of this paper is to effectively remove ECG artifacts from sEMG signals by a novel method.

**Methods:** In this paper, sEMG and ECG signals of the biceps brachii, brachialis, and triceps muscle of the human upper limb will be collected respectively. Firstly, an improved multi-layer wavelet transform algorithm is used to preprocess the raw sEMG signal to remove the background noise and power frequency interference in the raw signal. Then, based on the theory of blind source separation analysis, an improved Fast-ICA algorithm was constructed to separate the denoising signals. Finally, an ECG discrimination algorithm was used to find and eliminate ECG signals in sEMG signals. This method consists of the following steps: 1) Acquisition of raw sEMG and ECG signals; 2) Decoupling the raw sEMG signal; 3) Fast-ICA-based signal component separation; 4) ECG artifact recognition and elimination.

**Results and discussion:** The experimental results show that our method has a good effect on removing ECG artifacts from contaminated EMG signals. It can further improve the quality of EMG signals, which is of great significance for improving the accuracy of force estimation and motion intention recognition tasks. Compared with other state-of-the-art methods, our method can also provide the guiding significance for other biological signals.

## 1 Introduction

The past decade has seen the rapid development of Surface electromyogram (sEMG) in the biomedical field which is obtained from the surface of human skeletal muscle and reflects the changes of human nerves and muscles effectively. The essence of sEMG is a kind of nonlinear and non-stationary time series generated by the superposition of multiple muscles in time and space. Considering that sEMG has the characteristic of 30–150 ms ahead of body movement, hence, it is suitable for estimating muscle strength (Staudenmann et al., 2006; Tang et al., 2016). Furthermore, sEMG can also provide critical information about muscle rehabilitation and neurological dysfunction which has been widely used in sports biomechanics (Ye et al., 2015), and neuromuscular evaluation systems (Tang et al., 2018), prosthesis control (Castro et al., 2015), and other tasks. Evidence suggests that sEMG is an ideal control signal source among others.

sEMG signal is an important information source for human motion recognition and diagnosis. However, in the record of sEMG, the sensor is susceptible to heterogeneous noise (e.g., motion noise and Gaussian noise), cable movement, electrode-skin interface, and adjacent muscles. Therefore, the collected sEMG contains a large number of signal sources. What’s more, when some trunk muscles (e.g., bicep muscles, pectoral muscles, and back muscles) are located near the heart, the ECG signal produced by cardiac must be recorded together with the sEMG. This would make the sEMG contaminated and gets worse when the muscle gets closer to the cardiac (Allison, 2003).

To our knowledge, sEMG frequency band is between 20 Hz and 250 Hz, The frequency of the power signal is 50 Hz, ECG frequency band is between 0 Hz and 100 Hz. Due to the overlapping frequency distribution of these components, it would increase the power content of sEMG signal and distort the signal amplitude. Consequently, extracting effective information from sEMG signals becomes more and more difficult (Kuiken et al., 2004). Researchers show that when the muscle is in the state of complete relaxation, the sEMG signal is accompanied by signal pulsation, which can be determined to be the artifact generated by the periodic ECG signal (Joseph et al., 2007). In the EEG-FMRI experiment (Daly, 2021), the authors used a monopole lead to record the ECG signal, mainly because of the interference caused by the R wave in the ECG signal. In our previous work, we found that ECG artifacts have a great impact on the accuracy of elbow flexion force prediction (Lu et al., 2021). Therefore, it is crucial to improve the signal-to-noise ratio (SNR) of sEMG signal by removing the artifacts from sEMG (Abbaspour and Fallah, 2014). Currently, a considerable amount of literature has been published on signal processing research which has achieved good results (De Luca et al., 2010; Symeonidou et al., 2018). Some researchers pay attention to sEMG signal denoising (Nagasirisha B. and Prasad VVKDV., 2020; Lazaro et al., 2020). Other researchers have investigated ECG artifact removal. These methods can be classified into five general categories: High Pass based (HP-based), Template Subtraction based (TS-based), Wavelet-based (WT-based), Adaptive Filter-based (AF-based), and Blind Source Separation based (BSS-based). The characterization of these methods will be described in detail in Section 2.

In summary, the study of removing ECG artifacts from sEMG signals of upper limb muscles still faces the following challenges: 1. Some frequencies of the sEMG and ECG signals overlap, making it difficult to accurately remove ECG artifacts from the sEMG signal. 2. sEMG signals are susceptible to interference by factors such as muscle activity, electrode placement and individual differences, which increases the difficulty of ECG artifact removal. 3. It is difficult for current algorithms to balance the accuracy and real-time performance of artifact removal tasks. In recent years, our team has been committed to the research of perception and recognition of human muscle movement information, and has obtained certain results (Lu et al., 2021; Lu et al., 2022a; Lu et al., 2022b). The problem of ECG artifact removal in sEMG is the key to hindering our further research on human motion intention prediction and muscle force estimation tasks. Therefore, exploring the removal of ECG artifacts in sEMG is an important and meaningful work.

This paper aims at comprehensive research on eliminating ECG artifacts from sEMG signals of upper limb muscles based on the improved blind source separation method. We proposed a IWT-FastICA algorithm to realized the ECG artifact from sEMG. The study of using the IWT-FastICA algorithm represents a significant advancement in signal processing techniques. sEMG are often contaminated with artifacts caused by ECG signals, especially when the electrodes are placed close to the heart. These artifacts can obscure the underlying EMG activity, thus hindering accurate analysis and interpretation. The IWT is a powerful tool for signal decomposition and denoising. It breaks down the EMG signal into multiple layers, each representing a different frequency component. FastICA is an efficient independent component analysis (ICA) algorithm that separates mixed signals into their independent sources. In the context of EMG-ECG artifact removal, FastICA can be used to identify and isolate the ECG component from the mixed EMG signal. By combining IWT and FastICA, the IWT is first applied to the EMG signal to decompose it into multiple layers. Then, FastICA is used to identify and extract the ECG component from the decomposed layers. Finally, the extracted ECG artifact is subtracted from the original EMG signal, resulting in a cleaner EMG signal with reduced ECG interference. Our method was successfully applied to sEMG signals contaminated by ECG signals to eliminate ECG artifacts. The effectiveness of the proposed method is demonstrated by comparing the SNR, RE, and CC indexes with state-of-the-art methods.

## 2 Related works

According to the technology adopted, ECG artifact removal methods can be classified into five categories: High-pass Filtering based (HP-based), Template Subtraction based (TS-based), Wavelet Transform based (WT-based), Adaptive Filtering based (AF-based), Blind Source Separation based (BSS-based). Nevertheless, each method has its advantages and limitations. In this part, we will discuss the characteristics of each algorithm in detail.

### 2.1 High pass-based (HP-based)

According to the literature, Redfern et al. (1993) conclude that the increase of cutoff frequency can reduce the contamination of ECG signal and smooth the integrated signal. Ten years later, based on the finding of Redfern, Drake and Callaghan (2006) proved that a high-pass filter with a cutoff frequency of 30 Hz provides an optimal balance between ease of implementation, time commitment and performance. Although the high-pass filter can effectively remove low-frequency noise, it may also inhibit some useful low-frequency components in the signal, resulting in signal distortion. In addition, the cut-off frequency of the high-pass filter may be affected by environmental factors, making the filtering effect unstable.

### 2.2 Template subtraction-based (TS-based)

Template Subtraction technique is to remove artifacts by subtracting a template signal representing artifacts from the contaminated sEMG signal. In the literature, Abbaspour and Fallah (2014) firstly collected the sEMG signals, ECG artifacts, and ECG signals, then proposed an adaptive subtraction method to clean the contaminated sEMG signal. The relative error is 0.04 and the correlation is 97%. Limnuson et al. (2014) proposed an infinite impulse response (IIR) temporal filtering technique for real-time stimulus artifact rejection based on template subtraction. Junior et al. (2019) presented a template subtraction method for reducing electrocardiographic artifacts in sEMG signals which used the real contamination and emulated mixtures based on real signals. The above methods have preserved better the sEMG information compared with High-Pass Filtering based methods. The disadvantage of the TS-based technique is that it depends on the choice of template. If the template is not selected properly, it may lead to unsatisfactory filtering effect and even introduce new noise. In addition, the template subtraction filter is weak in processing dynamically changing signals, and it is difficult to adapt to the filtering requirements of non-stationary signals.

### 2.3 Wavelet-based (WT-based)

Wavelet decomposition is considered to be one of the most effective methods for processing non-stationary signals. The basic idea of ECG artifact removal by WT-based methods is based on the similarity of sEMG, ECG artifact, and wavelet function, then reset the wavelet coefficients and get a clean sEMG signal through wavelet reconstruction. Zhan et al. (2010) proposed a wavelet-based adaptive filter for removing ECG interference in EMGdi signals. Wavelet transform and ICA algorithm were combined to eliminate ECG artifacts (Taelman et al., 2007; Abbaspour et al., 2016) and proposed an ECG artifact removal algorithm combining wavelet with the ANFIS model. Luo and Yang (2018) presented a new algorithm to locate the peak value of ECG signal by using the square of the low-frequency coefficient and eliminate the ECG interference coefficient by using the “inverse” hard threshold. The disadvantage of wavelet transform is that it needs to decompose and reconstruct the signal at multiple levels. In addition, the performance of the wavelet filter is affected by the selection of wavelet basis function, and improper selection may lead to the degradation of the filtering effect.

### 2.4 Adaptive filter-based (AF-based)

Marque et al. (2005) proposed an adaptive filter method, which can effectively remove artifacts in the case of spectral overlap between sEMG signal and ECG signal. At the same time, this method can track the signal and noise in real-time. Qiu et al. (2015) found an adaptive matching filter based on a genetic algorithm, which can effectively extract sEMG from stimulated muscles and adjacent muscles. Nagasirisha B. and Prasad VVKDV (2020) proposed an Enhanced Squirrel Search (ESS) algorithm which is a combination of an adaptive Least Mean Square (LMS) filter and an adaptive Recursive Least Square (RLS) filter based on the adaptive filter. Compared with the mainstream methods, it eliminates noise and provides a noiseless sEMG signal to the output of the system. Using the Gram-Schmidt algorithm and the adaptive Prediction Error Filter (Yeom, 2005), Wang K. et al. (2020) described the design of a stimulus artifact removal system that operates at different frequencies, and the validity of the model was verified in healthy subjects with an average correlation coefficient of 0.94. However, The AF-based algorithm needs to adjust the filter parameters in real time according to the characteristics of the input signal to achieve the best filtering effect. When the input signal changes rapidly, it may be difficult for the adaptive filtering algorithm to converge quickly to the best state, resulting in a decline in filtering effect.

### 2.5 Blind source separation based (BSS-based)

Assumes that sEMG signal and ECG signal are independent of each other, BSS-based algorithms extract multiple independent components from the raw sEMG. The signal is purified by recognizing the information of the artifacts. Al Harrach et al. (2017) concluded a canonical component analysis-based technique to denoise HD-sEMG recordings at 20% of the maximum voluntary contraction. This research improved the signal-to-noise ratio (SNR) of sEMG signal in the isometric contraction task. Anand et al. (2018) proposed a method for artifact removal from the raw sEMG by using Canonical Correlation Analysis. Islam (2017) proposed an ECG mobility artifact removal algorithm based on ICA, which effectively realizes the artifact removal method without considering the mixed process information. Meanwhile, the results improve the accuracy by ∼9% in seizure detection and ∼24% in prediction. In the same year, Clarke et al. (2021) taking the advantage of the ICA and CCA, presented a novel denoising method called independent vector analysis (IVA). This method obtained better results in root mean square error and signal-to-noise ratio. In 2020, Schlink et al. (2020) summarized the different artifact removal algorithms and get the conclusion that the canonical correlation analysis filter is superior to the principal component analysis filter and high-pass filter in cleaning high-density sEMG during fast walking or running.

In summary, all the above methods could provide ECG removal with different effects. Nevertheless, each method has its limitation. Such of the defects of the high-pass filtering algorithm is that the low-frequency part of sEMG was also removed when the ECG is removed. The theoretical basis of the TS-based algorithm assumed that sEMG obeys Gaussian distribution with zero means, but this hypothesis is not satisfied in most situations. WT-based methods depict the two major flaws in the selection of mother wavelet and differentiation of model when the amplitudes of the source signal and artifact signal are similar. The main problem with AF-based methods is that it requires an extra sensor to record the clean ECG as a reference, which would increase the complexity of the algorithm. Hence, there is an urgent need for a better approach to ECG removal. Blind source separation technology can separate individual source signals from mixed signals without knowing the specific information of source signals in advance. Therefore, when the ECG artifact and sEMG tend to be mixed together, blind source separation technology can effectively separate them to achieve the removal of ECG artifact. Secondly, the sEMG signal is often interfered by various noises, but the blind source separation technology has strong adaptability to non-stationary signals and noisy environments, and can accurately separate the sEMG signal in complex environments, improving the quality and reliability of the signal.

## 3 Materials and methods

### 3.1 Component analysis of raw sEMG signal

In the field of biomedicine, artifacts refer to various forms of information that do not exist in the measured object but appear at the collection end. The generation of artifacts is mainly related to two factors: the condition of the subjects and measurement equipment. The existence of artifacts brings great difficulties to the analysis of raw signals. sEMG is physiological signals generated during muscle contractions and records the potential difference between two electrodes. The reference electrode is placed on the skin surface without muscle tissue. This will lead to the collected sEMG signal including all kinds of artifact components. Specifically, the artifacts can be divided into biological factors and technical factors (Wang HP. et al., 2020). Technical factors include inherent noise, 50 Hz power frequency interference, limb movement artifacts, and electromagnetic interference between devices. They can be removed by many effective denoising methods (Phinyomark, 2010; Gradolewski et al., 2015; Nagasirisha B. and Prasad VVKDV., 2020). However, biological factors are the specific attributes of the human muscle itself, which are mainly produced by cardiac, muscle movement and are hard to be removed fundamentally. It is worth noting that some artifacts can’t be avoided, but some can be controlled. Such as, the artifacts from the subjects and the equipment can be effectively suppressed in the experiment. However, for some artifacts that cannot be controlled, such as ECG signals, the method of threshold removal can be used to detect and eliminate them.

### 3.2 Principal of independent component analysis

Blind source separation (BSS) is to separate the independent signal source from the linear mixed observation signal by inferring the characteristics of the signal source. ICA is a branch of BSS. Based on high-order statistics of signals, ICA decomposes the independent components from the linear combination of several independent signal sources. BSS has been widely used in biomedical information processing (Flexer et al., 2005). The problem of blind source separation of signals is based on the assumption of statistical independence between the components of the observed mixed-signal $x\left(t\right)$ and the original signal $s\left(t\right)$ , and with the help of some prior knowledge of the probability distribution of the original input signal to recover the original input signal, eliminate the influence of the signal artifact, and realize the extraction of effective information in the original signal (Tong and Fei-Yun, 2016). Since the collected signals come from different signal sources, each original signal is considered to be independent of each other, that is, statistically independent signals from N signal sources are denoted as:

$$s_{1}\left(t\right),s_{2}\left(t\right)\ldots ,s_{n}\left(t\right)$$ (1)

Where, $s_{1}\left(t\right)$ represents first signal source, $s_{2}\left(t\right)$ represents the second signal source, $s_{n}\left(t\right)$ represents the *n*th signal source.

And the corresponding observed mixed signal is denoted as:

$$x_{1}\left(t\right),x_{2}\left(t\right),\ldots ,x_{n}\left(t\right)$$ (2)

Where, $x_{1}\left(t\right)$ represents the first observed mixed signal, $x_{2}\left(t\right)$ represents the second observed mixed signal, $x_{n}\left(t\right)$ represents the *nth* observed mixed signal.

Due to the mixed-signal having a linear characteristic and instantaneous characteristic, for i=1,2,…,n , the $x_{i}\left(t\right)$ can be drawn as:

$$x_{i}\left(t\right)=\sum_{j=1}^{n}a_{ij}s_{j}\left(t\right)$$ (3)

Where, $a_{ij}$ represents a coefficient or weight which is used to adjust or scale the amplitude of the signal $s_{j}\left(t\right)$ .

Hence, the sequence $x\left(t\right)$ can be expressed in vector and matrix form as:

$$x\left(t\right)=As\left(t\right)$$ (4)

where, $x\left(t\right)={\left[x_{1}\left(t\right),x_{2}\left(t\right),\ldots ,x_{n}\left(t\right)\right]}^{T}$ is the output signal observed through the sensor, $s\left(t\right)={\left[s_{1}\left(t\right),s_{2}\left(t\right)\ldots ,s_{n}\left(t\right)\right]}^{T}$ is the independent source of an unknown signal with zero means; $A=\left[\alpha_{ij}\right]\in R^{n\times m}$ represents the mixture matrix.

The separation signal can be calculated if we get the inverse matrix W of the mixing matrix A:

$$y\left(t\right)=Wx\left(t\right)$$ (5)

Where $y\left(t\right)$ is the separation signal which is equal to the independent sources of the unknown signal.

To sum up, the key to obtaining the separation signal $y\left(t\right)$ is to determine the separation matrix W , which needs to be determined by the independence measurement criterion between different signals.

### 3.3 Our proposed method

**FIGURE 1:**
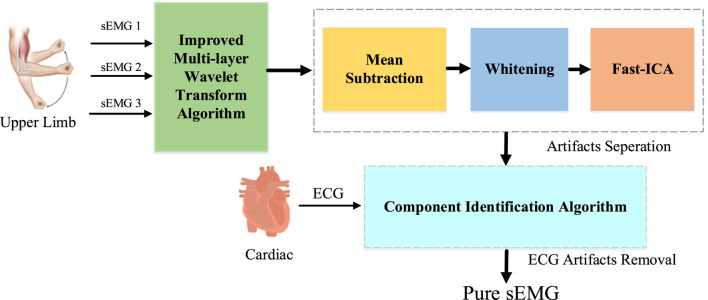
ECG artifact removal framework from sEMG signal.

sEMG sensors have multiple channel sensors which can simultaneously record the signals produced by “activity”, such as ECG, muscle movement, and cable movement. We assume that the source signals are statistically independent and non-Gaussian. Therefore, we use ICA based algorithm to separate the source signal into different components, then eliminate the unnecessary components. Finally, reconstruct the signal (Sheehan et al., 2022). The overall framework of our method is shown in Figure 1.

#### 3.3.1 Improved multi-layer wavelet transform algorithm for denoising

**FIGURE 2:**
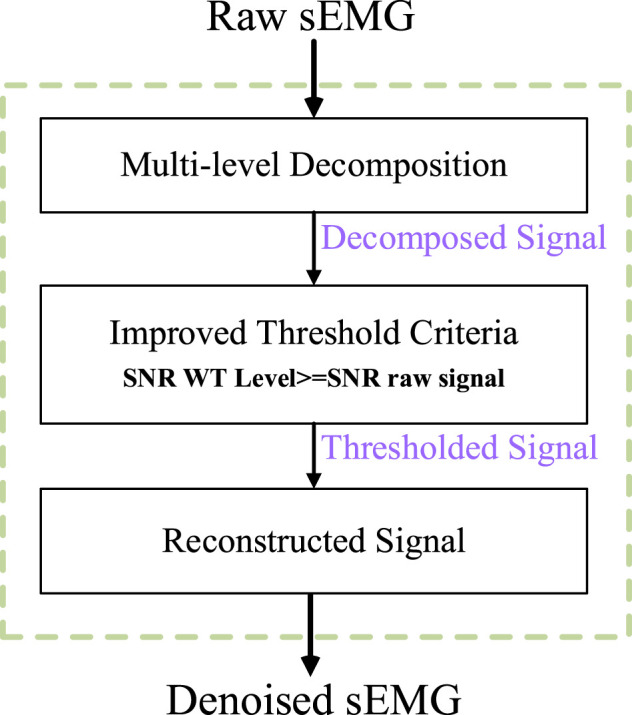
Block diagram of multi-layer wavelet transform for sEMG denoising.

Typically, sEMG signals are non-stationary signals contaminated by various noises generated by skin-electrode interfaces, electronic devices, and external sources. Therefore, before effectively removing ECG artifacts of sEMG signals, appropriate filtering procedures should be used to purify the signals. The wavelet transforms denoising method has been proposed in the early years and has got good results. In this study, we adopt a new wavelet transform denoising method based on multi-layer decomposition analysis (El hanine et al., 2020; Li et al., 2021). The emphasis of this method is to select a new threshold rule for sEMG reconstruction and denoising. The flowchart of the algorithm is shown in Figure 2. The process can be divided into three steps, namely, *multi-level decomposition*, *thresholding criteria,* and *reconstructed signal.*

The Wavelet Transform (WT) of the input sEMG signal is expressed as:

$$W_{a,b}\left(t\right)=\int sEMG\left(t\right)\Psi *\left[\left(t-b\right)/a\right]dt\;a\neq 0$$ (6)

Where $W_{a,b}\left(t\right)$ represents wavelet coefficient, a and b represent the scale parameters and the shift parameters, is the time shift, $sEMG\left(t\right)$ represents the original signal, Ψ (t) represents the wavelet basis function, it must satisfy mathematical criteria like finite energy and no zero-frequency component to be admissible which is defined as:

$$\Psi_{a,b}\left(t\right)=\frac{1}{\sqrt{a}}\Psi \left(\frac{t-b}{a}\right)$$ (7)

Step 1: *Multi-level decomposition*. The raw sEMG time series is decomposed by a pair of finite impulse response filters, which is represented by a low-pass filter and a high-pass filter. The low-pass filter is to extract the approximation coefficients ( $y_{low}$ ), and the high-pass filter is to get the detail coefficients ( $y_{high}$ ). The filter outputs are then down-sampled as given by Eqs 8, 9, respectively. It is worth noting that, the 4th order with 9 levels of decomposition wavelet Daubechies is performed.

$$y_{low}=\sum_{k=-\infty }^{\infty }x\left[k\right]*g\left[2n-k\right]$$ (8)

Where, $y_{\text{low}}$ is the low-pass component of the output signal which represents the result obtained after the input signal passes through the low-pass filter g, $x\left[k\right]$ represents the sample value of input signal at time *k,* $g\left[2n-k\right]$ represents the coefficient or response of the low-pass filter g at time *2n-k.*

$$y_{high}=\sum_{k=-\infty }^{\infty }x\left[k\right]*h\left[2n-k\right]$$ (9)

Where, $y_{high}$ represents the high-pass component of the output signal which represents the result obtained after the input signal x passes through the high-pass filter h, $x\left[k\right]$ represents the sample value of input signal at time *k,* $h\left[2n-k\right]$ represents The coefficient or response of the high-pass filter h at time *2n-k*.

**FIGURE 3:**
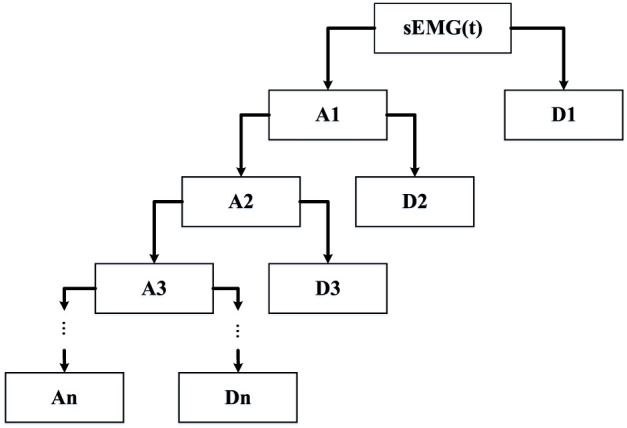
The diagram of the multi-layer wavelet.

Step 2: *Threshold Criteria*. Considering the information contained in each detail, the threshold criteria of each level are established. The higher the frequency, the greater the correlation between detail and noise. What’s more, to obtain clean surface sEMG signals, the signal-to-noise ratio index is associated with each wavelet transform layer. When the WT level associated with SNR is higher than the SNR ratio of the original signal, the decomposition level was selected. The diagram of multi-layers wavelet denoising is shown in Figure 3. As can be seen from the diagram, A1, A2, A3 … , An is the approximates of composition in different scales, and D1, D2, D3, … , Dn are the details of composition in different scales.

The key to affecting the effect of denoising is the selection of threshold function and quantifying wavelet coefficients according to thresholds. Traditional threshold functions are divided into the hard threshold and soft threshold functions which can be described by the following equation (He et al., 2023).

$$w_{j,k}^{\prime }=\left\{\begin{array}{c} w_{j,k}\;\left|w_{j,k}\right|\geq \lambda \\ 0\;\left|w_{j,k}\right|\leq \lambda \end{array}\right.$$ (10)

$$w_{j,k}^{\prime }=\left\{\begin{array}{c} sign\left(w_{j,k}\right)\times \left(\;\left|w_{j,k}\right|-\lambda \right)\;\left|w_{j,k}\right|\geq \lambda \\ 0\;\left|w_{j,k}\right|\leq \lambda \end{array}\right.$$ (11)

Nevertheless, there are some shortcomings of these two threshold functions. First of all, the hard threshold function is discontinuous in the whole wavelet domain and discontinuous at the point of ±λ . Secondly, there is a constant deviation between $w_{j,k}$ and $w_{j,k}^{\prime }$ in the soft threshold function. Consequently, we proposed an improved threshold function that can overcome these shortcomings. It can be described as follows:

$$w_{j,k}^{\prime }=\left\{\;\begin{array}{c} w_{j,k}-\frac{2\lambda }{1+\exp\left(\lambda -w_{j,k}\right)}\;w_{j,k}\geq \lambda \;\\ 0\;\left|w_{j,k}\right|<\lambda \\ w_{j,k}+\frac{2\lambda }{1+\exp\left(\lambda +w_{j,k}\right)}{\;w}_{j,k}<-\lambda \end{array}\right.$$ (12)

The following conclusions can be drawn from Eq. 12:

1. As the value of $w_{j,k}$ approaches ∞ , the deviation between $w_{j,k}$ and $w_{j,k}^{\prime }$ would disappear.
2. The function meet that the noise coefficient decreases while the signal coefficient increases in the wavelet domain.
3. When the $w_{j,k}$ closes to the threshold ±λ , $w_{j,k}^{\prime }$ is going to approach zero and make the function continuous.

Step 3: *Reconstructed Signal*. Applying this threshold criterion application, the new detail coefficients and the original approximation coefficients are used to reconstruct the denoised sEMG signal.

#### 3.3.2 Mean subtraction and whitening

To facilitate calculation, the observed vector x should be preprocessed. We convert the observed vector x to the intermediate output Z , which mainly includes meaning subtraction and whitening. Mean Subtraction is subtracting its mean from the observed vector. That is, transform the observation vector (Dong et al., 2020; Jayasanthi et al., 2020). $x\left(t\right)$ into zero mean vector which can be represented by the following formula:

$$x^{\prime }=x-\mu$$ (13)

Where, $x^{\prime }$ represents the vector with zero mean after mean subtraction, x represents the observed vector, μ represents the expectation of the observed vector.

In general, the collected data are correlated, so it is necessary to whiten the data to remove the correlation between observation signals and simplify the extraction process of independent components. Furthermore, the convergence of the algorithm is better after whitening. The new data $x^{\prime }$ satisfies two properties after whitening: 1. The correlation between features is low. 2. All the features have the same variance. The description of the algorithm is as follows steps:

Step 1. We defined the input sample data as:

$${{Χ}^{\prime }=x}^{\prime }\in R^{n\times m}$$ (14)

Where, n represents the dimension of data, m represents the number of samples.

Step 2. Calculate the covariance matrix after mean subtraction ${Χ}^{\prime }$ :

$$C_{X^{\prime }}=\frac{1}{m}\sum_{i=1}^{m}\left[{Χ}^{\prime }{{Χ}^{\prime }}^{T}\right]$$ (15)

Step 3. The covariance matrix is decomposed by eigenvalue decomposition:

$$C_{X^{\prime }}=U\Lambda U^{T}$$ (16)

Step 4. Rotate the data:

$$X_{rot,i}=U^{T}x$$ (17)

Step 5. Scale the data on each principal component axis so that its variance is 1.

$$X_{PCAwhiten,i}=\frac{X_{rot,i}}{\sqrt{\lambda_{i}}}$$ (18)

From what has been discussed above, Principal Component Analysis (PCA) whitening is defined as:

$$X_{PCAwhiten}=\Lambda^{-\frac{1}{2}}U^{T}X=\left[\begin{array}{ccc} \frac{1}{\sqrt{\lambda_{1}}} & \cdots & 0\\ \vdots & \ddots & \vdots \\ 0 & \cdots & \frac{1}{\sqrt{\lambda_{n}}} \end{array}\right]X_{rot,i}$$ (19)

After PCA whitening, the covariance matrix of data is an identity matrix, that is, each dimension becomes irrelevant and the variance of each dimension is 1.

#### 3.3.3 Improved Fast-ICA

Fast-ICA, also known as a fixed-point algorithm, takes the maximum negative entropy as a search direction. The independent sources are extracted sequentially, and the fixed-point iterative optimization algorithm is adopted to make the convergence of the results faster and more robust.

Fast-ICA aimed to find an optimal direction w which makes the non-Gaussian property of this direction maximize: $\max J\left(w^{T}x\right)$ (Qiu et al., 2022; Koldovsk et al., 2022).

In this paper, we selected the negative entropy to measure non-Gaussian which is defined as:

$$J\left(w^{T}x\right)={\left[E\left\{G\left(w^{T}x\right)\right\}-E\left\{G\left(v\right)\right\}\right]}^{2}$$ (20)

Where v represents the random variable with zero mean unit variance. $G\left(∙\right)$ represents any non-quadratic function, we select the $G\left(w^{T}x\right)=\tanh\left(w^{T}x\right)$ .

The source signal is estimated by maximizing the objective function, and the approximate maximum of negative entropy $J\left(w^{T}x\right)$ is generally obtained at the extreme value of $E\left\{G\left(w^{T}x\right)\right\}$ . Based on the Lagrange condition, we can find the extreme value of $E\left\{G\left(w^{T}x\right)\right\}$ under the constraint condition of ${\left\|w\right\|}^{2}=1$ .

$$E\left\{xg\left(w^{T}x\right)\right\}+\beta w=0$$ (21)

Where $g\left(∙\right)$ is the derivative $G\left(∙\right)$ .

Here we used the Newton iteration method to solve the problem of finding the roots of the equation which can be expressed as:

$$x_{n+1}=x_{n}-\frac{f\left(x_{n}\right)}{f^{,}\left(x_{n}\right)}$$ (22)

The iteration formula used by Fast-ICA can be described as:

$$\left\{\begin{array}{c} w_{n+1}=E\left\{xg\left(w^{T}x\right)\right\}-E\left\{g^{\prime }\left(w^{T}x\right)\right\}w_{n}\\ w_{n+1}=\frac{w_{n}}{\left\|w_{n}\right\|} \end{array}\right.$$ (23)

As we all know, the initial values of Fast-ICA are chosen randomly, different initial values would affect the accuracy and convergence of the algorithm. Currently, the optimal method to reduce the sensitivity of the algorithm is by using the fastest descent method. It is an optimization algorithm that takes the negative gradient direction as descending direction. The improved algorithm can be described as follows:

(1) Firstly, construct a random matrix: w .
(2) Calculate the gradient value of $E\left\{xg\left(w^{T}x\right)\right\}$ at w .
(3) Calculate the iteration step λ .
(4) Update the iteration equation based on the faster descent method:

$$w_{n+1}=w_{n}+\lambda E\left\{xg\left(w^{T}x\right)\right\}$$ (24)

#### 3.3.4 ECG component identification algorithm

The different components obtained by the improved Fast-ICA algorithm are considered to be the multiple signal sources. Therefore, the active components of the ECG need to be accurately identified. In information theory, entropy is used to evaluate the degree of signal confusion. Compared with sEMG signals, ECG signals are used to represent the rhythm and activity of the human heart which have more regular, less complex, and lower entropy characteristics. Hence, artifact components can be separated effectively by the entropy criterion. Compared with approximate entropy and sample entropy, fuzzy entropy has the advantages of better anti-noise performance and higher stability (Erdal et al., 2023). Therefore, fuzzy entropy is used as the basis for ECG component discrimination. The calculation process is as follows:

1. Let the time series with length N be denoted as $\left\{X\right.\left(i\right)\left.,i\epsilon \left(1,n\right)\right\}$ .
2. With the size of the sliding window as M, a group of M-dimensional vectors are generated in sequence and denoted as:

$$X_{i}^{m}\left(t\right)=\left\{x\left(i\right),x\left(i+1\right),\ldots ,x\left(i+m-1\right)\right\}-x_{0}\left(t\right)$$ (25)

$$x_{0}\left(t\right)=\frac{1}{m}\sum_{j=0}^{m-1}x\left(i+j\right)$$ (26)

3. Calculate the distance $d_{ij}^{m}$ between any two vectors, which is the maximum value of the difference between the two corresponding elements:

$$d_{ij}^{m}=\max_{k\in \left(0,m-1\right)}\left\{\left|\left.x\left(i+k\right)-x_{0}\left(i\right)]-[x\left(j+k\right)-x_{0}\left(j\right)\right]\right|\right\}$$ (27)

4. The degree of similarity between sequences is calculated by the fuzzy membership function, which is defined as:

$$A\left(x\right)=\left\{\begin{array}{l} 1\;x=0\\ \exp\left[-\left(\ln2\right){\left(\frac{x}{r}\right)}^{2}\right]\;x>0 \end{array}\right.$$ (28)

Where, r is the similarity tolerance parameter, which is defined as r times the standard deviation of one-dimensional time series.

Therefore, the similarity between the two vectors $X_{i}^{m}\left(t\right)$ and $X_{j}^{m}\left(t\right)$ is:

$$D_{ij}^{m}=\exp\left[-\left(\ln2\right){\left(d_{ij}^{m}/r\right)}^{2}\right]$$ (29)

5. Define function:

$$\Phi^{m}\left(t\right)=\frac{1}{N-m+1}\sum_{i=1}^{N-m+1}\frac{1}{N-m}\sum_{j=1,j\neq i}^{N-m+1}D_{ij}^{m}$$ (30)

6. Increase the sliding window M to M+1 and repeat steps from (2) to (5), obtained $\Phi^{m+1}\left(t\right)$

Therefore, the fuzzy entropy can be calculated as:

$$FuzzyEn\left(t\right)=\ln\Phi^{m}\left(t\right)-\ln\Phi^{m+1}\left(t\right)$$ (31)

7. Finally, ECG components in sEMG can be identified by threshold judgment. In this paper, the threshold discriminant proposed by Tichavsk et al. (2006) is used as threshold judgment. The detailed description is as follows:

$$\Phi \left(k+1\right)-\Phi \left(k\right)<\Phi \left(k\right)-\Phi \left(k-1\right)$$ (32)

Where, $\varphi \left(k\right)$ represents the entropy value of the Kth independent component after ascending order.

If the k value is satisfying the above formula, k is the minimum integer satisfying the conditions, and the Fast-ICA components corresponding to the first K entropy values are determined as ECG artifacts and eliminated.

## 4 Experimental analysis

### 4.1 Design of experimental

**FIGURE 4:**
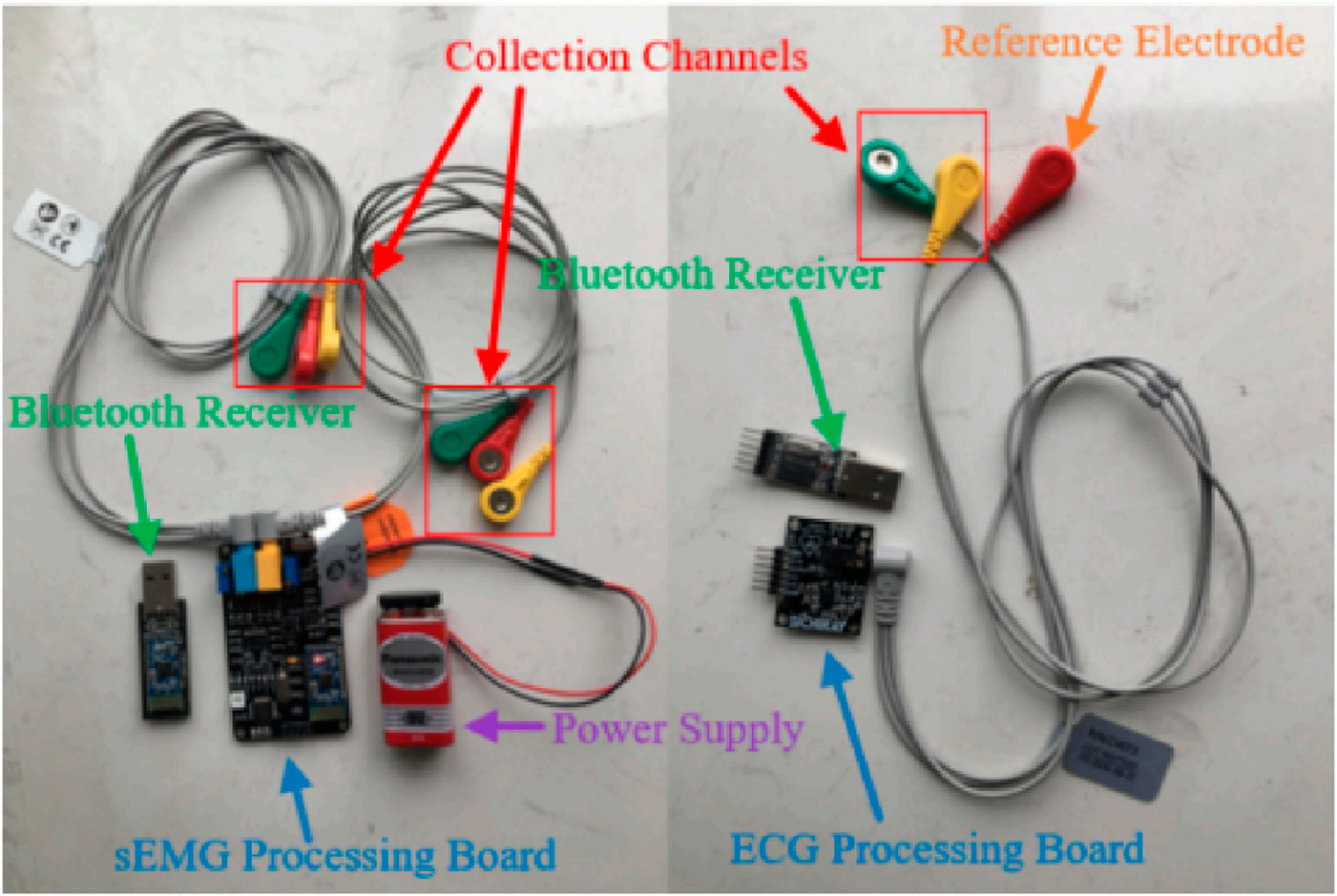
Illustration of sEMG and ECG wireless acquisition system.

**FIGURE 5:**
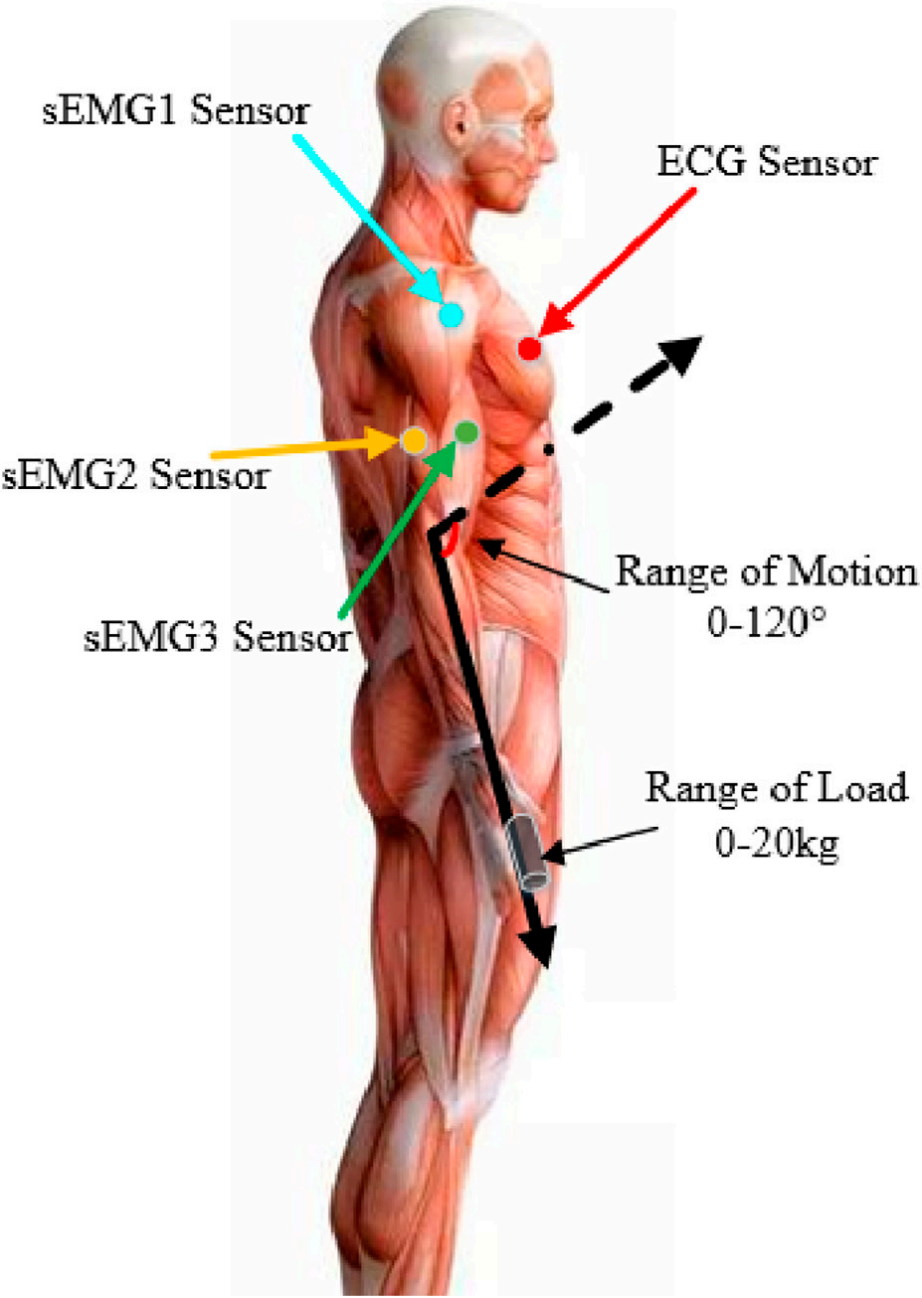
Schematic diagram of the subject experiment.

**FIGURE 6:**
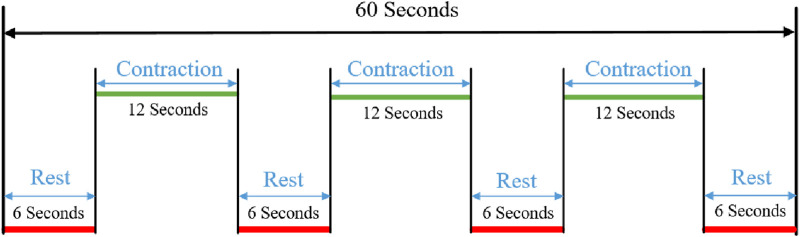
Signal acquisition mechanism.

In this experiment, we selected five healthy adult subjects without musculoskeletal diseases or a history of major upper limb injuries. Three sEMG sensors were placed on the different muscles of the upper limb to collect the raw sEMG signal. Meanwhile, the ECG sensor was placed on the chest of each subject. Each subject grabbed a different mass of the load to perform isokinetic contraction to highlight the validity of the experimental results. All participants have informed the consent of the experiment procedure and signed the informed consent form. The physical parameters of the subjects are shown in Table 1. The Illustration of sEMG and ECG wireless acquisition system and the schematic diagram of the subject experiment are shown in Figures 4, 5. The detailed parameters of ECG sensor and sEMG sensor are shown in Table 2. Each subject performed three contraction tasks, and the muscles were fully rested before each contraction. The experiment lasted for 60 s in total. The experimental scheme adopted in this paper is shown in Figure 6.

**TABLE 1:** Physical parameters of each subject.

	Subject 1	Subject 2	Subject 3	Subject 4	Subject 5
Age (years)	26	23	28	30	22
Sex (M/F)	M	M	M	F	F
Weight (kg)	77	78	75	55	51
Height (cm)	176	180	173	162	160
BFR (%)	19.6%	18.1%	20.3%	26.6%	23.6%

**TABLE 2:** Detailed description of ECG Sensor and sEMG sensor.

Parameters	ECG sensor	sEMG sensor
Model	ADS1293	EDK0056
Functional description	• 3 channel, 24 bit analog front end	• High sensitivity, capable of capturing weak EMG signals
	• Low noise and low power consumption	• Used for surface EMG acquisition
	• Built-in programmable gain amplifier	
Power supply voltage	DC (5V)	DC (5V)
Development environment	IAR Embedded Workbench	IAR Embedded Workbench
Temperature range	−20°C∼85°C	−20°C∼60°C
Sampling frequency	Up to 25.6 ksps	depends on the configuration
Input range	±400 mV	Covers the typical range of EMG signals
Input noise	7μVpp (40 Hz bandwidth)	—
Output signal	Analog Signal	Analog Signal
Communication	SPI	Bluetooth 4.0

### 4.2 sEMG signal denoising and performance analysis

**FIGURE 7:**
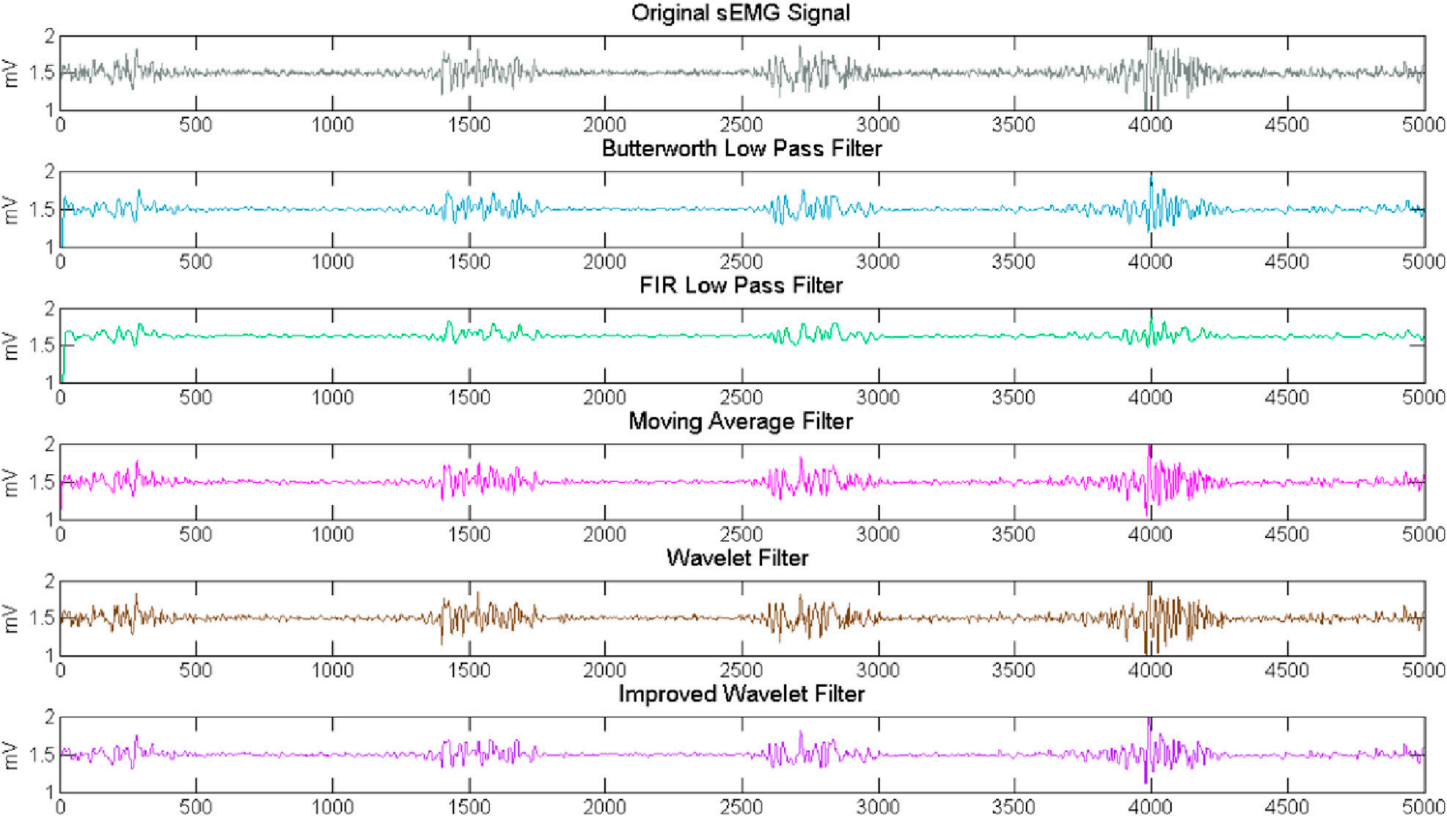
Comparison results of different filter.

We compare the effectiveness of five mainstream denoising algorithms. They are Butterworth low-pass filtering algorithm (Barin and Zencir, 2022), FIR low-pass filtering algorithm (Yang et al., 2022), moving average filtering algorithm (Lee, 2014) and wavelet filtering algorithm (Ramakrishnan and Selvan, 2003). The filtering results are shown in Figure 7. From the figure, we can see that the Butterworth filter, Moving Average filter and Improved Wavelet filter got better performance. To further verify the effectiveness and superiority of the denoising methods, correlation coefficient (CC) and Root Mean Square Error (RMSE) indicators are chosen to evaluate the performance of the different algorithms (Yoo et al., 2018; Iluore et al., 2022). The results are detailed in Table 3.

$$CC=\frac{Cov\left(x,y\right)}{\sqrt{Var\left(x\right)}∙\sqrt{Var\left(y\right)}}$$ (33)

Where, $Cov\left(x,y\right)$ is the covariance of x and $y;\;Var\left(x\right)$ is the variance of $x;Var\left(y\right)$ is the variance of y .

$$RMSE={\left(N^{-1}\sum_{i}{\left(S^{\prime }\left(i\right)-S\left(i\right)\right)}^{2}\right)}^{1/2}$$ (34)

Where, $S^{\prime }\left(i\right)$ represents the denoised value; $S\left(i\right)$ represents the original value.

**TABLE 3:** The comparison of performance indicators.

	Butterworth	FIR	Moving average	Wavelet	Improved wavelet
CC	0.911	0.891	0.915	0.902	**0.924**
RMSE	4.885	5.323	4.784	5.121	**4.693**

The bold values represent statistically significant levels.

Our proposed denoising method gets the best performance in two indexes. The CC reflects the integrity of valid information retention, the larger the CC is, the higher correlation between the raw signal and the filtered signal. RMSE reflects the difference between the raw signal and the filtered signal.

### 4.3 ECG artifact removal results

**FIGURE 8:**
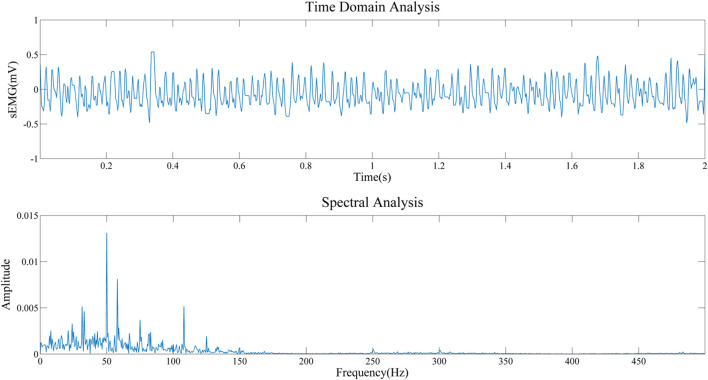
Frequency domain analysis of sEMG after filtering in resting state.

**FIGURE 9:**
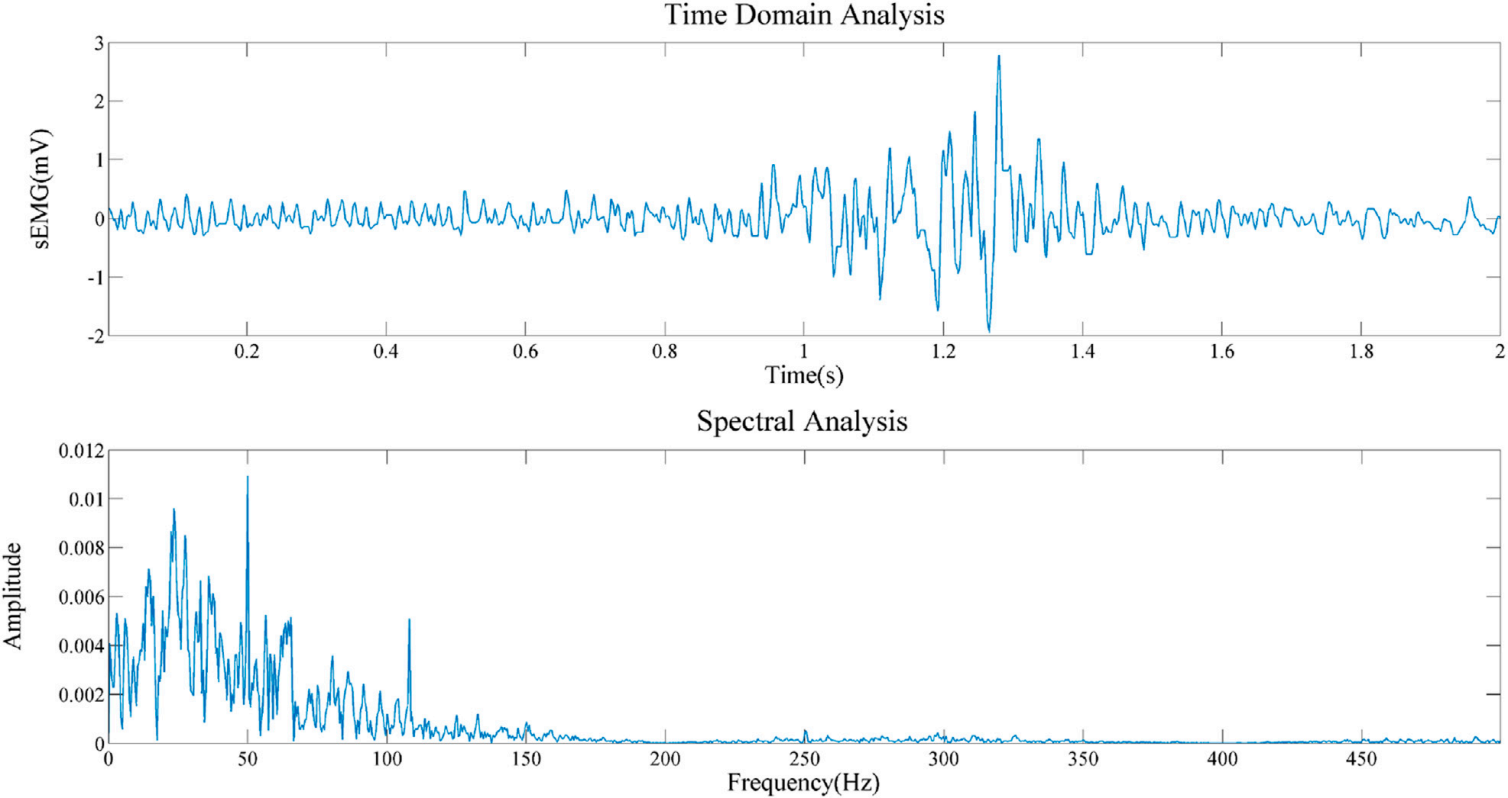
Frequency domain analysis of sEMG after filtering in contractile state.

In order to prove the effectiveness of the next method, we collected the EMG of biceps muscle in static and dynamic conditions, and analyzed the frequency domain information after filtering algorithm, as shown in Figures 8, 9. It can be seen that the frequency distribution of the filtered EMG signal is 0 ∼ 500 hz, mainly concentrated in 0 ∼ 150 hz; 2. In both cases, there will be obvious spikes around 50 Hz and 100 Hz. In addition to the power supply interference factor, the largest factor is caused by the ECG artifact, because the frequency of the signal is close to the power supply frequency or its frequency multiplier, which appears as a spike in the frequency domain plot.

**FIGURE 10:**
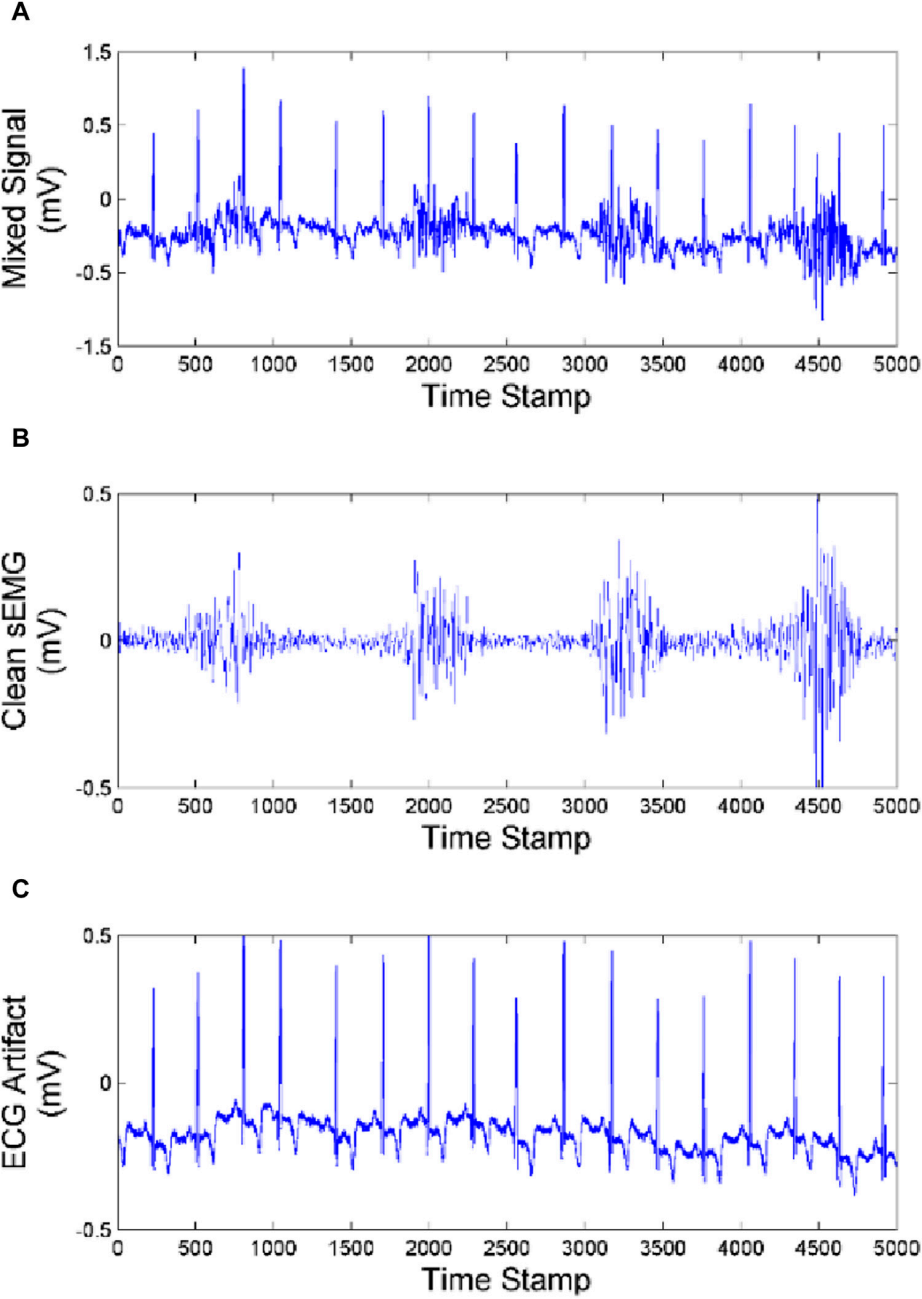
Effect of artifact removal of biceps brachii **(A)** Mixed Signal. **(B)** Clean sEMG signal. **(C)** ECG artifact.

**FIGURE 11:**
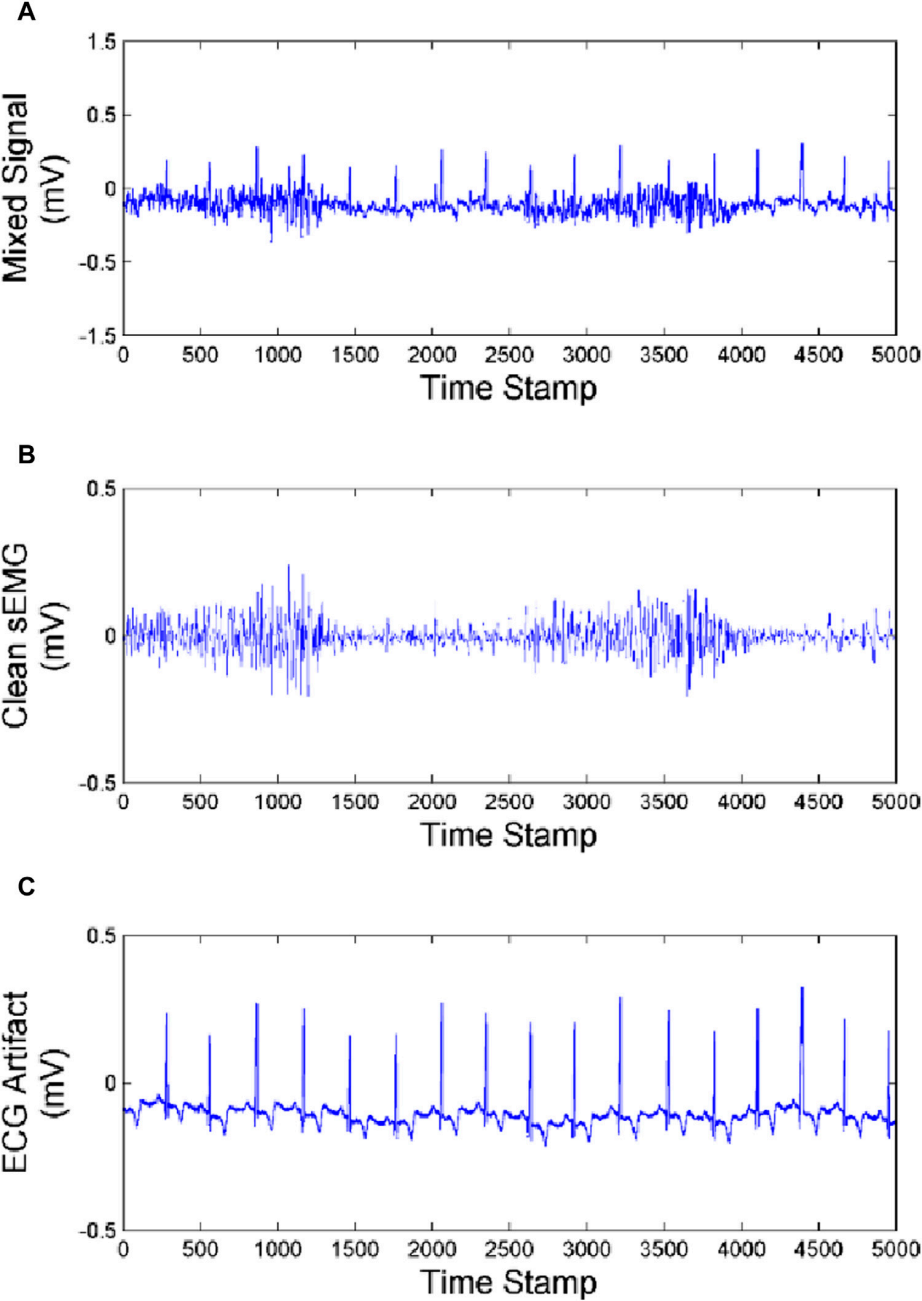
Effect of artifact removal of triceps **(A)** Mixed Signal. **(B)** Clean sEMG signal. **(C)** ECG artifact.

**FIGURE 12:**
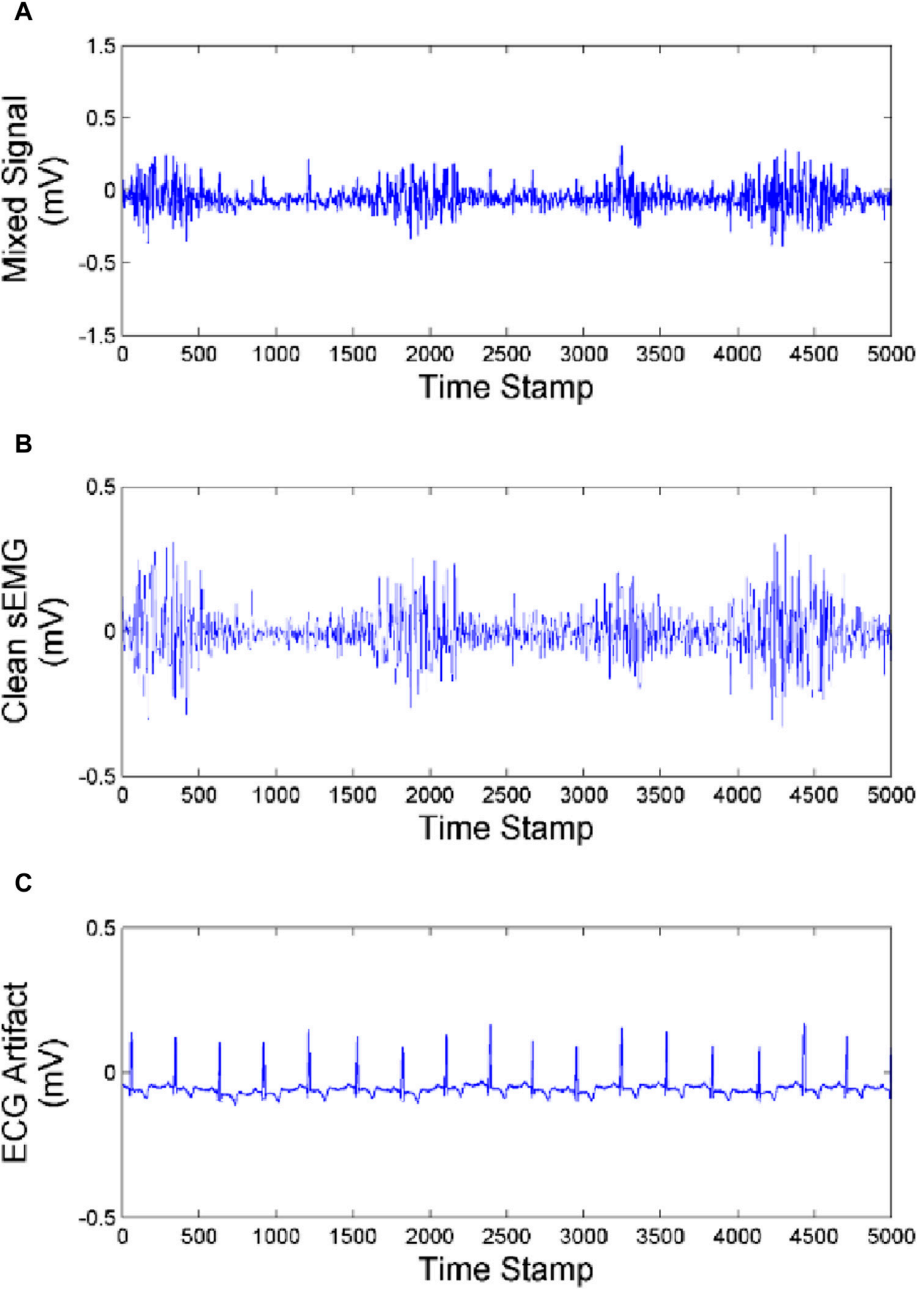
Effect of artifact removal of brachialis **(A)** Mixed signal. **(B)** Clean sEMG signal. **(C)** ECG artifact.

In order to clarify the effectiveness and universality of our artifact removal algorithm, we analyzed the experimental results of one subject and compared the ECG artifact removal results from the biceps brachii, triceps and brachialis as shown in Figures 10–12.

As we can see from Figures 10–12, the muscles closer to the cardiac are more affected by the ECG. Muscles farther from the heart are less affected by ECG. Our method achieves good performance in removing ECG artifacts in sEMG signal.

**FIGURE 13:**
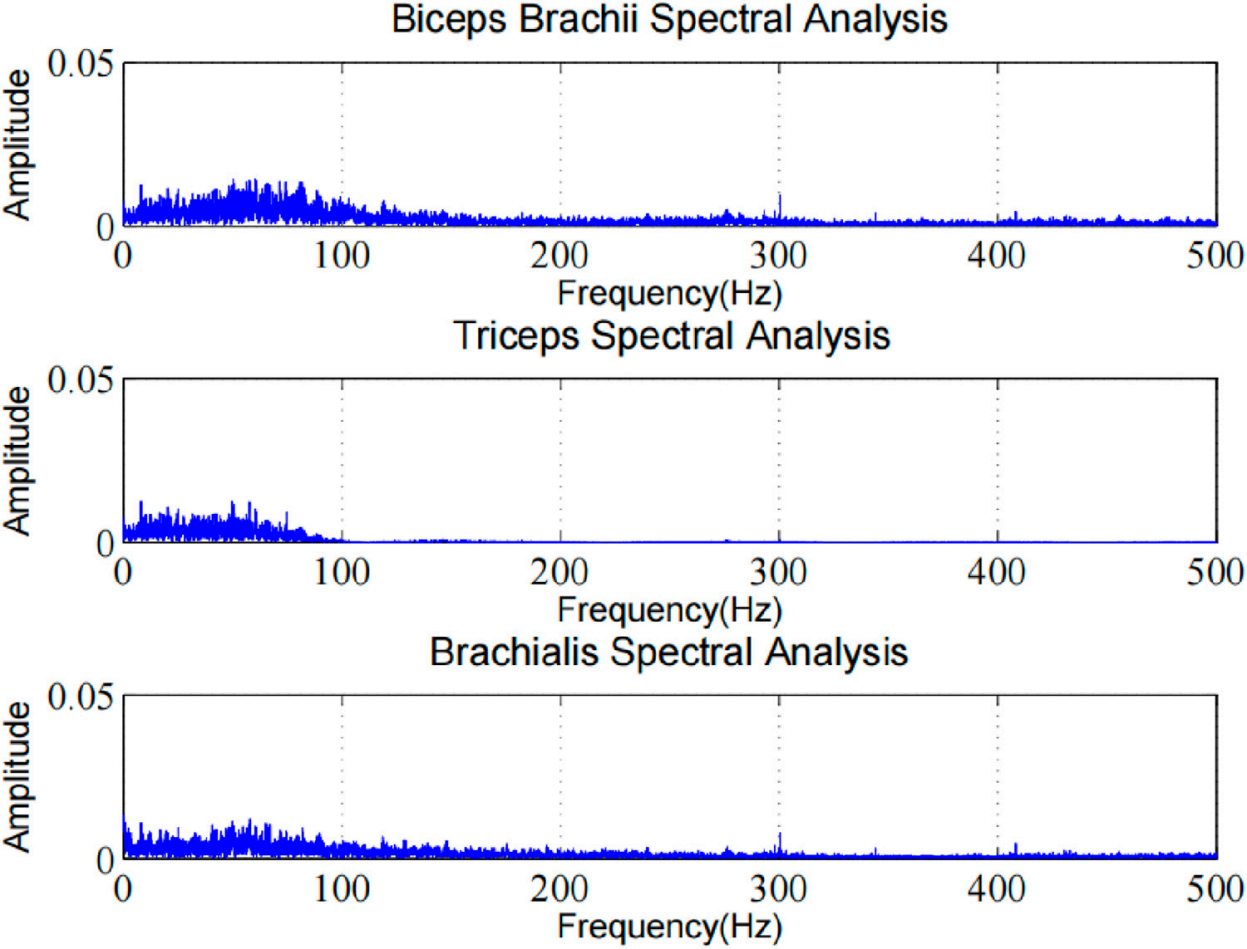
Frequency domain analysis of ECG artifacts in different muscle regions.

In order to fully demonstrate the advantages of the proposed algorithm in ECG artifact removal, we performed a frequency-domain analysis and explored the ECG artifact removal effectiveness for different muscle locations. As shown in Figure 13.

The experimental results clearly show the ECG artifact removal performance at different muscle positions. In frequency domain analysis, we observed that our method can effectively identify and separate ECG artifact components. By comparing the spectra before and after processing, it can be found that the characteristic frequencies of ECG artifacts are significantly suppressed, while the useful frequency components of sEMG signals are well preserved. This result proves that our algorithm has good artifact removal ability in frequency domain, and can effectively reduce the interference of ECG artifact to sEMG signal analysis. The algorithm shows good artifacts removal effect at different muscle positions, and retains the original features of sEMG signal.

To further evaluate the performance of the algorithm, we made statistics on the experimental results of five subjects respectively, and calculated Relative Error (RE), CC(Correlation Coefficient) and Signal-to-Noise Ratio (SNR) indicators in sEMG signals, as shown in Table 4. Compared with the state-of-the-art methods, the results are shown in Table 5.

**TABLE 4:** The evaluation indexes of each subject and their mean.

	RE	CC (%)	SNR
Adult 1	0.07	92.8	11.35
Adult 2	0.09	97.2	10.27
Adult 3	0.12	96.4	10.56
Adult 4	0.08	97.5	8.92
Adult 5	0.07	93.1	9.96

**TABLE 5:** Comparison of Artifact Removal performance of different methods.

	HP-based	TS-based	WT-based	AF-based	Ours
RE	0.06	0.08	0.07	0.08	0.09
SNR	7.19	7.56	9.45	8.75	10.23
CC	92.67%	94.98%	96.28%	95.45%	97.56%

We compare the current mainstream algorithms with the methods in this paper in terms of accuracy, efficiency and calculation requirements, as shown in Tables 6–8. It is worth noting that the actual performance indicators of each algorithm are affected by the differences in design parameters, hardware implementation methods and input signal characteristics. The specific performance of each algorithm indicator will vary according to the actual situation. Therefore, we have only combined many experiments with a general understanding and cannot cover all possible situations.

**TABLE 6:** Efficiency performance comparison.

	Computing efficiency level (1–10)	Pros	Cons
HP-based	High (8)	Simple implementation, can quickly filter out low-frequency components	Some useful information will be lost
TS-based	Medium (5)	The realization is simple and the calculation speed is fast	Sensitive to template selection and may introduce errors
WT-based	High (8)	Multi-scale analysis for non-stationary signals	Sensitive to the selection of wavelet base and the computational complexity may be high
AF-based	Medium to high (5–8)	Automatic adjustment of filter parameters to adapt to signal changes	The initial rate of convergence may be slow
Ours	High (8)	Fast independent component analysis for multi-channel signals	The statistical characteristics of the signal are required

**TABLE 7:** Accuracy performance comparison.

	Computing accuracy level (1–10)	Features
HP-based	Medium (5)	Suitable for removing low frequency noise and retaining high frequency signal
TS-based	Medium (5)	Suitable for removing background noise and extracting dynamically changing targets
WT-based	High (8)	It has multi-resolution characteristics and is suitable for non-stationary signal processing
AF-based	High (8)	The filter parameters can be automatically adjusted according to the characteristics of the input signal, which is suitable for the occasions where the statistical characteristics of noise are unknown
Ours	High (8)	Based on the negative entropy maximization criterion, it has the characteristics of good robustness, fast convergence and high precision

**TABLE 8:** Computing requirements comparison.

	Computational complexity	Computation amount	Real-time performance	Memory requirements
HP-based	Medium (5)	Medium (5)	Preferably (7)	Medium (5)
TS-based	Lower (3)	Lower (3)	Preferably (7)	Lower (3)
WT-based	Preferably (7)	Preferably (7)	Preferably (7)	Preferably (7)
AF-based	Preferably (7)	Preferably (7)	Preferably (7)	Medium (5)
Ours	Lower (3)	Medium (5)	Medium (5)	Lower (3)

It can be seen from Tables 4, 5, the CC index and SNR index of the proposed method perform best, indicating that the sEMG signal after artifact removal in this paper contains more effective information. At the same time, the algorithm can effectively improve the quality of the signal while removing artifacts, and make the useful information in the signal more prominent.

## 5 Conclusion

A new artifact removal algorithm in sEMG combining an improved multi-layer wavelet transform algorithm and Fast ICA algorithm is presented, which is called the IWT-FastICA algorithm. By using improved multi-layer wavelet transform techniques, improved threshold criteria, and through a large number of comparison experiments, our proposed method has a better performance in the signal denoising aspect when compared with other state-of-art filters. For a signal with ECG artifacts, we adopt FastICA, which has the best performance in a blind separate source algorithm. Furthermore, the application of fuzzy entropy theory improves the recognition rate of ECG artifacts. By the experimental analysis of different muscles and different movements, the ECG artifacts caused by heart beating can be significantly improved. The experimental results show that there are significant improvements in objective indicators and real application.

The results of this study can provide effective guidance for human-computer interaction and robot compliant control and optimization of biomechanical models. In addition, compared with other mainstream methods, the proposed method has some advantages, but it still has some limitations in practical application, such as performance degradation and high calculation cost when processing complex signals. Therefore, the combination of deep learning algorithm and signal processing to achieve the effective removal of ECG artifacts is worthy of further exploration. We will focus on the following aspects to carry out further research work: 1. Combined with noise suppression technology which can remove artifacts and reduce the influence of other noises on sEMG signal. 2. Combination with feature extraction and classification algorithm, more useful information can be extracted from the processed sEMG signal and some tasks (such as action recognition and muscle force estimation) can be completed. 3. Integration of cross-domain technologies: learn from advanced technologies in other fields (such as signal processing, pattern recognition, machine learning, etc.) to further improve the performance of the algorithm.

## Data Availability

The original contributions presented in the study are included in the article/supplementary material, further inquiries can be directed to the corresponding author.
